# Supplementary material for: Chromatin enrichment for proteomics in plants (ChEP-P) implicates the histone reader ALFIN-LIKE 6 in jasmonate signalling
Source: BMC Genomics. 2021 Nov 22;22:845. doi: 10.1186/s12864-021-08160-6 (PMC8609783; doi:10.1186/s12864-021-08160-6)
Supplement: Supplementary file 1 — Additional file 1: Supplemental Fig. S1. [file 12864_2021_8160_MOESM1_ESM.pdf]

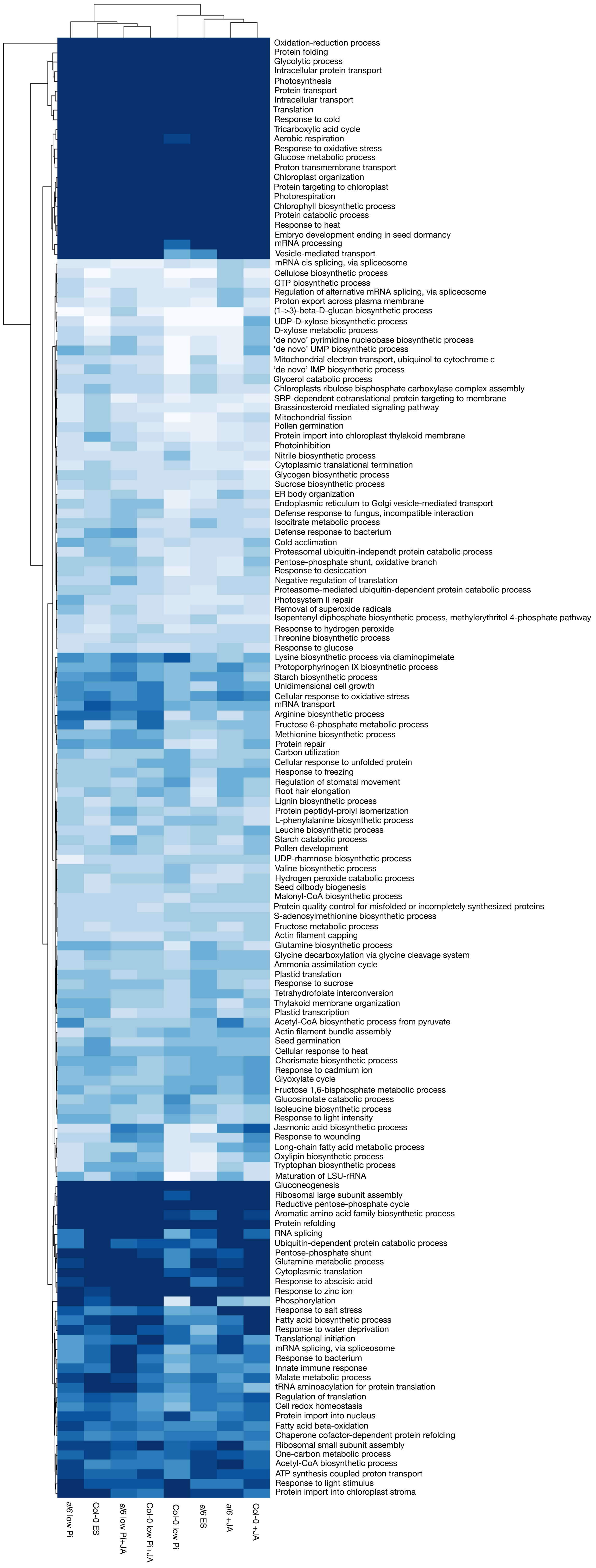

Figure S1. Extended GO analysis of proteins identified by ChEP-P. GO enrichment was computed by TopGO using the elim method (Alexa et al., 2006) by implementation of GOBU (https://gobu.sourceforge.io/). Heatmap was generated with the pheatmap package in R. $P = 0.00001$.
