# Supplementary material for: Chromatin enrichment for proteomics in plants (ChEP-P) implicates the histone reader ALFIN-LIKE 6 in jasmonate signalling
Source: BMC Genomics. 2021 Nov 22;22:845. doi: 10.1186/s12864-021-08160-6 (PMC8609783; doi:10.1186/s12864-021-08160-6)
Supplement: Supplementary file 2 — Additional file 2: Supplemental Fig. S2. [file 12864_2021_8160_MOESM2_ESM.pdf]

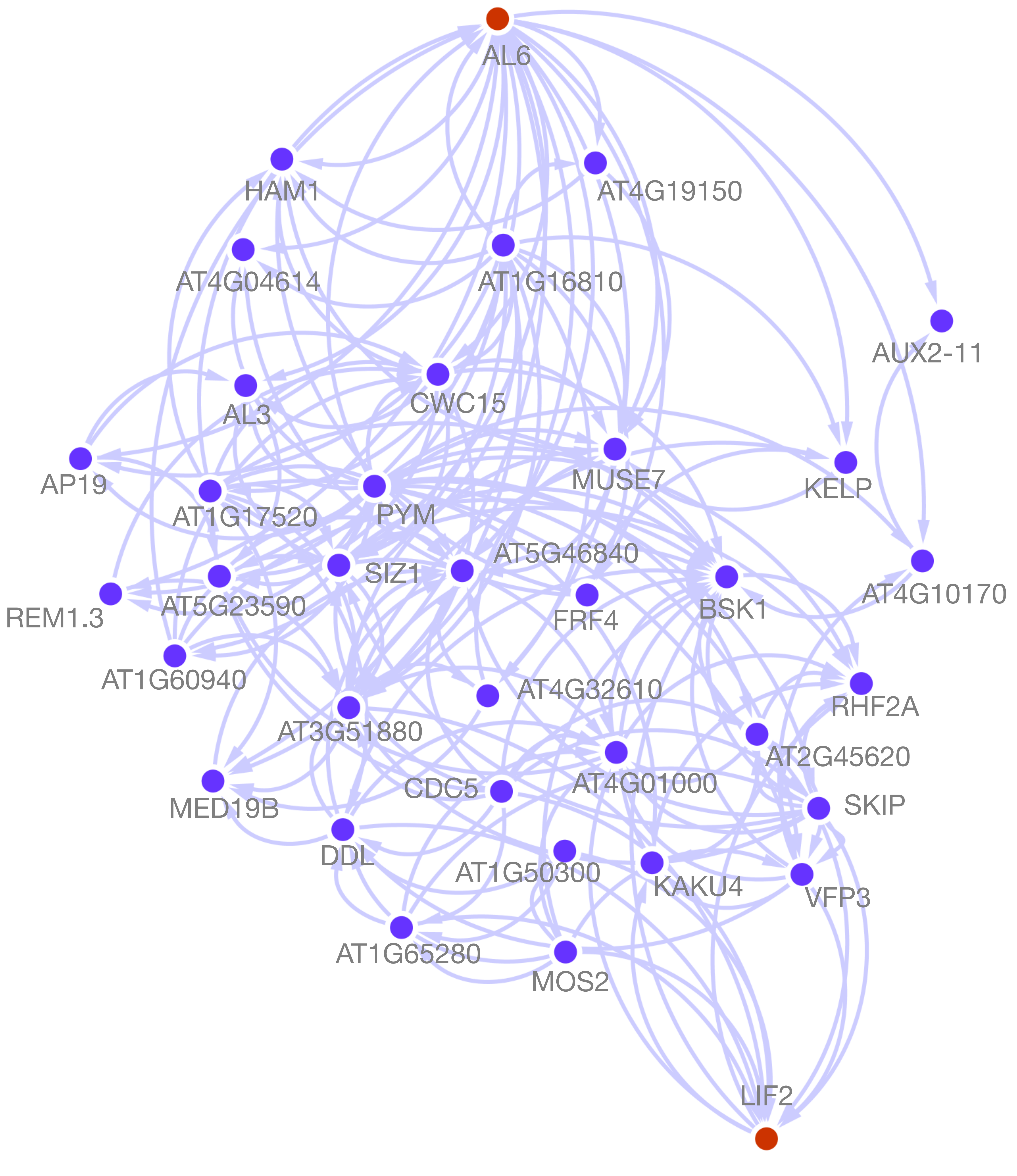


**Figure S2.** Seedling-specific co-expression network of AL6 and LIF2. Co-expression network around *AL6* and *LIF2* in Arabidopsis seedlings. A total of 5,556 RNA-seq data sets were downloaded from the Sequence Read Archive (SRA) hosted by the National Center for Biotechnology Information (NCBI) and normalized. Networks were constructed with the MACCU toolbox (Lin et al., 2011) based on pair-wise comparison of the co-expression relationships of Arabidopsis genes expressed in seedlings with a Pearson coefficient > 0.6 using seedling-specific data sets.
