# Supplementary material for: Chromatin enrichment for proteomics in plants (ChEP-P) implicates the histone reader ALFIN-LIKE 6 in jasmonate signalling
Source: BMC Genomics. 2021 Nov 22;22:845. doi: 10.1186/s12864-021-08160-6 (PMC8609783; doi:10.1186/s12864-021-08160-6)
Supplement: Supplementary file 3 — Additional file 3: Supplemental Fig. S3. [file 12864_2021_8160_MOESM3_ESM.pdf]

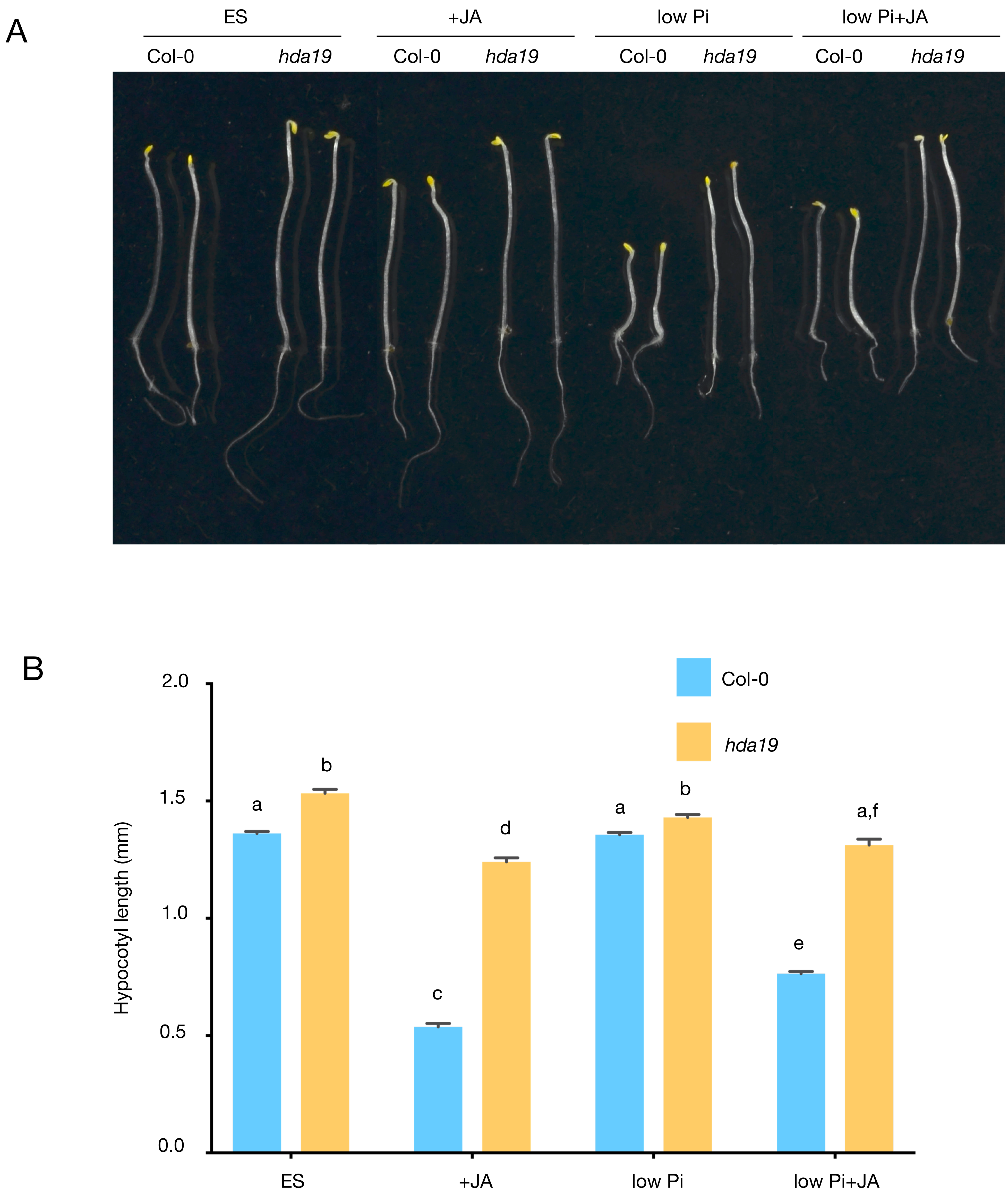

Figure S3. Phenotype of etiolated *hda19* mutant seedlings in response to various treatments. A, Five-day-old Col-0 (wild type, WT) and *hda19* seedlings on mock (ES) medium, or media supplemented with 50 μM JA (+JA), 2.5 μM Pi (low Pi), or 2.5 μM Pi + 50 μM JA (low Pi+JA) in darkness. B, Quantification of hypocotyl length. Three independent experiments with $n \geq 60$ were performed. Error bars represent SE. Letters above bars indicate significant differences ($P < 0.01$) as determined by two-way ANOVA with Tukey test using multiple comparison between cell means regardless of row and columns. GraphPad Prism 8.0 was used to generate graphs and to conduct statistical analysis.
